# Refuge or predation risk? Alternate ways to perceive hiker disturbance based on maternal state of female caribou

**DOI:** 10.1002/ece3.2672

**Published:** 2017-01-06

**Authors:** Frédéric Lesmerises, Chris J. Johnson, Martin‐Hugues St‐Laurent

**Affiliations:** ^1^ Département de Biologie, Chimie et GéographieCentre for Northern StudiesUniversité du Québec à RimouskiRimouskiQCCanada; ^2^ Ecosystem Science and Management ProgramUniversity of Northern British ColumbiaPrince GeorgeBCCanada

**Keywords:** activity budget, ecotourism, human shield, linear features, maternal state, *Rangifer*, space use

## Abstract

Human presence in natural environments is often a source of stress that is perceived by large ungulates as an increased risk of predation. Alternatively, disturbance induced by hikers creates a relatively predator‐free space that may serve as a refuge. We measured the behavioral responses of female caribou to disturbance associated with the presence of hikers during summer in the Gaspésie National Park. We used those data to determine whether caribou responded negatively to human activity (i.e., the *predation risk hypothesis*) or whether human activity resulted in a decrease in the magnitude of perceived risk (i.e., the *refuge hypothesis*). Female caribou with a calf spent nearly half of their time feeding, regardless of the presence of a trail or the number of hikers. They also decreased their vigilance near trails when the number of hikers increased. Conversely, lone females fed less frequently and almost doubled the time invested in vigilance under the same circumstances. However, both groups of females moved away from trails during the day, especially in the presence of hikers. We demonstrated that risk avoidance was specific to the maternal state of the individual. Lactating females accommodated the presence of hikers to increase time spent foraging and nutritional intake, providing support for the refuge hypothesis. Alternatively, lone females with lower energetic requirements and no maternal investment in a vulnerable calf appeared less tolerant to risk, consistent with the predation risk hypothesis. *Synthesis and applications*: Hikers influenced the vigilance–feeding trade‐off in caribou, underlining the importance of appropriate management of linear structures and human activities, especially across the critical habitat of endangered species. Even if some individuals seemed to benefit from human presence, this behavioral adaptation was not sufficient to reduce annual calf mortality associated with predation.

## 1 Introduction

Human disturbance can be an important driver of animal behavior (Ciuti et al., 2012; McLoed, Guay, Taysom, Robinson, & Weston, 2013). Humans and their activities may be perceived as predation risk (i.e., *predation risk* hypothesis) (Frid & Dill, 2002) leading to a landscape of fear and the altered distribution or behavior of wildlife species (Hernandez & Laundré, 2005; Laundré, Hernandez, & Ripple, 2010; Rösner, Mussard‐Forster, Lorenc, & Müller, 2014) with the potential for fitness costs (Dussault, Pinard, Ouellet, Courtois, & Fortin, 2012; Strasser & Heath, 2013). In some instances, habitat alteration and the stress associated with human presence have led to dramatic decreases in population distribution and abundance (Brooks et al., 2002; Krauss et al., 2010). Even within protected areas, where often there are conservation measures designed to maintain natural and undisturbed habitats for wildlife, most animals must cope with a variety of anthropogenic disturbances (e.g., roads, resorts, cabins, and hiking trails) (Brown et al., 2012; Richard & Côté, 2015).

Across many landscapes, humans often play the role of the apex predator and shape prey and predator distributions (Basille et al., 2009; Valeix, Hemson, Loveridge, Mills, & Macdonald, 2012). Unlike predators, however, humans may be more spatiotemporally predictable, especially in parks, where hunting is often prohibited and visitation most often occurs during daytime and on roads and hiking trails. In such an environment, habituation to human‐caused threat is a possibility (Bremset‐Hansen & Aanes, 2015). Alternatively, outside parks where hunting is allowed, humans may act as the dominant natural predator and an important driver of ungulate behavior (Ciuti et al., 2012). In many cases, large carnivores are much less adaptable to human presence. They may adjust their movement to the spatiotemporal pattern of human activity, avoiding anthropogenic features when humans are present (Theuerkauf, Jedrzejewski, Schmidt, & Gula, 2003; Hebblewhite & Merrill, 2008; Ordiz, Stoen, Delibes, & Swenson, 2011; Valeix et al., 2012). This may occur even in parks where human activities are highly regulated (Whittington, St. Clair, & Mercer, 2005). In such cases, prey can modulate predation risk by exploiting the enemy‐free spaces created when the presence of humans and associated infrastructure displaces predators (i.e., the *refuge hypothesis*) (Berger, 2007; Shannon, Cordes, Hardy, Angeloni, & Crooks, 2014; Steyaert et al., 2016). Predation risk varies in space and time, and thus, antipredator strategies should be adaptable to variation in risk. In protected areas, prey should adjust their space use to match the diurnal pattern of human activity, such as hikers on marked trails during the day.

Even when selecting habitat with lower predation risk, prey are rarely completely safe (Elgar, 1989; Lima & Dill, 1990). Thus, an animal's activity budget is often a trade‐off between security and food intake (Fortin, Boyce, Merrill, & Fryxell, 2004), which is mainly influenced by the risk associated with the occupied habitat (Boving & Post, 1997; Gavin & Komers, 2006; Liley & Creel, 2008) and by the quality of food resources (Fortin & Fortin, 2009). Also, intrinsic factors such as age, body condition, and reproductive status could interact to influence the trade‐off between vigilance and foraging (Bachman, 1993; Wolff & Van Horn, 2003; Winnie & Creel, 2007).

For mammals, lactation has an extremely high energetic cost; females often adjust their behavior, including exposure to risk, to meet those nutritional demands (White & Berger, 2001; Wolff & Van Horn, 2003; Hamel & Côté, 2008). Where nutritional intake is limited, lactating females might be more tolerant of human activities that reduce predation risk and increase foraging time (Lima & Bednekoff, 1999). According to the two main hypotheses that relate behavior to the trade‐off between nutrition and risk, lactating females should tolerate low‐risk disturbances and use human presence as a refuge against predation to reduce vigilance and increase the time spent foraging (i.e., the *refuge hypothesis*). In contrast, females without a calf should be less prone to take risk, adjusting their vigilance to human presence (i.e., the *predation risk hypothesis*).

In this study, we assessed the behavioral response of Atlantic‐Gaspésie caribou (*Rangifer tarandus caribou* Gmelin, hereafter referred to as Gaspésie caribou) to the presence of hikers in the Gaspésie National Park. We used those data to determine whether variation in human activity triggered a vigilance response associated with risk, the predation risk hypothesis, or a decrease in the magnitude of perceived risk, the refuge hypothesis. We tested those hypotheses relative to the maternal state of individual caribou: females with and without a dependent calf.

## 2 Material and Methods

### 2.1 Study area

**Figure 1:**
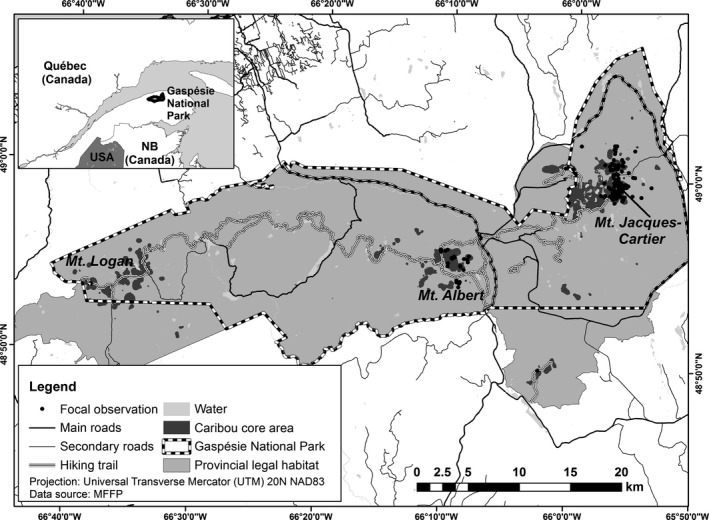
Estimated location of each focal caribou during behavioral observations in Gaspésie National Park, during the summers of 2013–2014

The study area covered the range of the Gaspésie caribou population, corresponding approximately to the limit of the Gaspésie National Park (48°50′N; 66°00′W) and surrounding habitat protected by provincial law (Figure 1). The caribou range encompasses the McGerrigle Mountains, in its eastern part, which are dominated by Mount Jacques‐Cartier (1,268 m), and the Chic‐Chocs Mountains in the western part, which include Mount Albert (1,154 m) and Mount Logan (1,128 m). The altitudinal gradient determines three ecological zones characterized by differences in vegetation type. The highest elevation zone (>1,050 m) is alpine tundra and is characterized by a mat of lichens, mosses, and graminoids along bare rocks and ericaceous shrubs. The subalpine forest (900–1,050 m) is essentially a transition zone where tree height decreases with altitude, forming a Krummholz belt before transitioning to alpine tundra. Finally, the montane area (100–900 m) is represented by closed forest composed of balsam fir (*Abies balsamea* Mill.), white spruce (*Picea glauca* Moench), black spruce (*P. mariana* Mill.), and birch (*Betula* sp.). Most caribou are found at elevations >700 m and are subdivided into three subpopulations, namely Albert (*n *= ~15 individuals), Jacques‐Cartier (*n *= ~70 individuals), and Logan (*n *= ~15 individuals) (Ouellet, Ferron, & Sirois, 1996; Mosnier, Ouellet, Sirois, & Fournier, 2003).

Gaspésie National Park is mostly visited during summer months, especially during July and August (L. Sirois & G. Fortin, unpublished data). Some of the most popular trails pass through critical habitat for caribou (Figure 1). To decrease human access during periods when caribou are vulnerable to disturbance, access to these trails is closed from October to mid‐June. Buses, available from 10:00 to 16:00, are also required to access the “Jacques‐Cartier” trail. Moose (*Alces americanus americanus* Gray), black bears (*Ursus americanus* Pallas), coyotes (*Canis latrans* Say), and a few white‐tailed deer (*Odocoileus virginianus* Zimm.) are also found within Gaspésie National Park. Wolves (*Canis lupus* L.) were extirpated from the south shore of the St. Lawrence River in the mid‐1800s.

### 2.2 Caribou locations

To test the two competing hypotheses, we used GPS collars to collect spatial locations for Gaspésie caribou. In total, 43 adult caribou (17 M; 26 F), proportionally distributed among the three subpopulations (i.e., McGerrigle: *n *= 28; Albert: *n *= 6; Logan: *n *= 9), were captured, fitted with GPS‐Argos telemetry collars and followed for 2.5 years. Collars were programmed to acquire locations every two (model TGW‐4680‐3, Telonics Inc. Mesa, AZ, USA) or 3 hr (model TGW‐4680, Telonics Inc. Mesa, AZ, USA) and to transfer relocations from the past week via an Argos link every 4 days. To limit the potential negative impacts of helicopter activity, and as recommended by the Animal Welfare Committee [Université du Québec à Rimouski (UQAR) certificate #CPA‐52‐13‐112; ministère des Forêts, de la Faune et des Parcs (hereafter MFFP) certificate #CPA FAUNE 13‐08], captures were divided into two sessions of 22 and 21 animals each, conducted in early winter 2013 and 2014, respectively. For these analyses, we only considered location data collected for females and categorized individuals according to the presence of a calf (Table 1). We calculated the mean proportion of locations for each 100‐m distance class of a trail for closed and open hours. Caribou were found mostly in open alpine areas resulting in a low probability of habitat‐related bias in GPS fix success. Also, we did not assess any behavioral or location parameters that could be influenced by fix rate. Thus, for these analyses, we used all the location data, regardless of fix interval.

**Table 1:** Presence of calf during focal observation of female caribou in Gaspésie National Park, during the summer tourism period (25th May–20th August)

ID—Subpop.	2013			2014		
	With calf	Without calf	No. of GPS locations	With calf	Without calf	No. of GPS locations
CG02—McG	25th May	4th July	372	25th May	15th July	176
CG04—McG	–	25th May	877	–	–	
CG05—McG	25th May	–	300	–	25th May	121
CG06—McG	25th May	26th June	867	–	–	
CG11—Alb	25th May	26th May^a^	306	–	25th May	296
CG13—McG	25th May	–	204	–	25th May	308
CG16—Alb	25th May	‐	408	–	25th May	324
CG20—McG	–	25th May	659	–	25th May	135
CG23—McG	25th May	–	978	25th May^b^	–	225
CG25—McG	–	–		25th May	3rd July	251
CG27—McG					25th May	245
CG30—McG	–	–		–	25th May	174
CG37—McG				–	25th May	350
CG40—McG	–	–		–	25th May	359
CG41—McG	–	–		25th May	4th June	333
CG42—McG	–	–		–	25th May	275
CG43—McG	–	–		–	25th May	365
CG45—McG	–	–		–	25th May	392

We indicated the first date of each maternal state, by year, for all females followed during this study. CG25 to CG45 were captured in February 2014. CG04 and CG06 died during winter 2013–2014.

a We did not do any focal observation before CG11 lost its calf. CG11 was considered as without a calf in 2013.

b CG23 died with its calf by predation in 16 June 2014.

### 2.3 Activity budget

We conducted 30‐min focal observations of collared females exclusively for the Mount Albert and McGerrigle subpopulations during the summer tourism period (25th May–20th August). We restricted our observations to these two subpopulations because it was difficult to access and observe caribou on Mount Logan. To find caribou, we walked along the two main hiking trails (Jacques‐Cartier and Albert) watching for females. We also used VHF and GPS collar locations to locate caribou when they were not visible from the trails. We stayed as far as possible from caribou during the focal observations. When more than one collared female was located, we randomly chose one animal and described its behavior according to 12 activity budget categories: lying (lying on ground, regardless of the head position), feeding (standing head down, including the time biting, cropping, and masticating), walking, food searching (walking head down, looking for food), running, trot, vigilance (standing and being alert, ears pointing in a specific location), standing (no alert position, often ruminating), grooming, social interaction, and other (represented less than 0.5% of the observation period). We noted the individual's identification (confirmed with VHF frequency and ear tag), time of day, date, group size, and the approximate distance and the azimuth of the caribou from our observation point. We stopped the focal observation if the caribou detected our presence.

### 2.4 Estimating disturbance by hikers

We deployed 23 trail cameras (Spypoint BF‐6, GG Telecom, Victoriaville) on hiking trails and monitored human presence. Most of the trails had two cameras, one at the beginning and one almost on the summit. Each trail was divided into 200‐m segments and we calculated the number of hikers per hour on each segment, assuming a constant speed of hikers between each camera. We related each caribou observation to the nearest trail segment and inferred the number of hikers that occurred at that time (rounded to the nearest hour) and place.

In addition to the number of hikers, we related caribou behavior to hourly temperature using meteorological stations installed on three summits in the National Gaspésie Park (L. Sirois & G. Fortin, unpublished data). For each focal observation, we used the air temperature at the nearest meteorological station.

### 2.5 Geomatics analyses

Using a GIS database including trails and habitat categories (derived from a 1: 20,000 ecoforestry map, MFFP), we calculated the distance of the observed caribou to the nearest trail and the percentage of open habitat in a 200 m radius from the caribou location. Minimum mapping unit size was 4 ha for forested polygons and 2 ha for nonforested areas (e.g., water bodies, bare rock). Open habitat (2‐ha resolution) included alpine tundra and wetlands. In the model below, we developed a distance variable that was tested as a decay function with different constants [exp(‐α/distance), where α = 50, 100, 250, or 500] (Carpenter, Aldridge, & Boyce, 2010) or as a binary variable (0 < threshold > 1) with different thresholds (100, 250, and 500 m). The decay or binary method providing the lowest AIC_*c*_ (Burnham & Anderson, 2004) was retained and used in our subsequent statistical analyses. For time spent feeding, the most parsimonious covariate for distance to a trail was a decay distance (α = 250) and a binary variable (0 ≤ 500 m > 1) for female without and with a calf, respectively. For time spent vigilant, the most parsimonious distance covariates were binary with thresholds of 100 and 500 m for females without and with a calf, respectively. Although more complex, a unique decay function or distance threshold allowed us to better represent the behavioral response of each demographic group.

### 2.6 Statistical analyses

We focused the statistical analyses exclusively on the vigilance and foraging behaviors of monitored caribou. A literature review suggested that these were the most important behaviors for testing the trade‐off between security and food acquisition as well as the propensity of females to use human presence as a refuge against predation (Wolff & Van Horn, 2003; Winnie & Creel, 2007). We used a negative binomial distribution to model the number of seconds spent in vigilance during the focal observation and a fractional logit regression (sensu Papke & Wooldridge, 1996) to assess the proportion of the activity budget spent feeding. For both analyses, we used the individual ID as a random factor to take into account interindividual variability in behavior (Gillies et al., 2006). We used AIC_*c*_ to evaluate the importance of hikers relative to the foraging and vigilance behavior of observed caribou. We fit two statistical models for each behavior, testing whether the addition of the anthropogenic variables (i.e., distance to a trail, number of hikers, and the interaction between them) to our basic model (i.e., group size, temperature in °C as a continuous variable, hour as a continuous variable, and proportion of open habitat) resulted in a more parsimonious model. We normalized all independent variables, except time of day, for better model convergence. We used cross‐validation to assess the predictive ability of the most parsimonious model. We fitted the model with 80% of the data and then performed a Spearman correlation (*r*_*s*_) between predicted and observed values for the independent data (20%).

We used Kolmogorov–Smirnov tests to compare space use by caribou along trails. We compared the frequency distribution of caribou locations at four distances from the nearest trail (>4,000, 3,000, 2,000, and 1,000 m) relative to opening hours (trails closed or opened) and the presence of hikers (hikers vs. no hikers). This resulted in 12 comparisons. Habitat characteristics do not change along trails according to the time of day or the presence of hikers; thus, such covariates were unnecessary for this analysis. All statistical analyses were conducted using R (The R Core Team version 2.15).

## 3 Results

**Figure 2:**
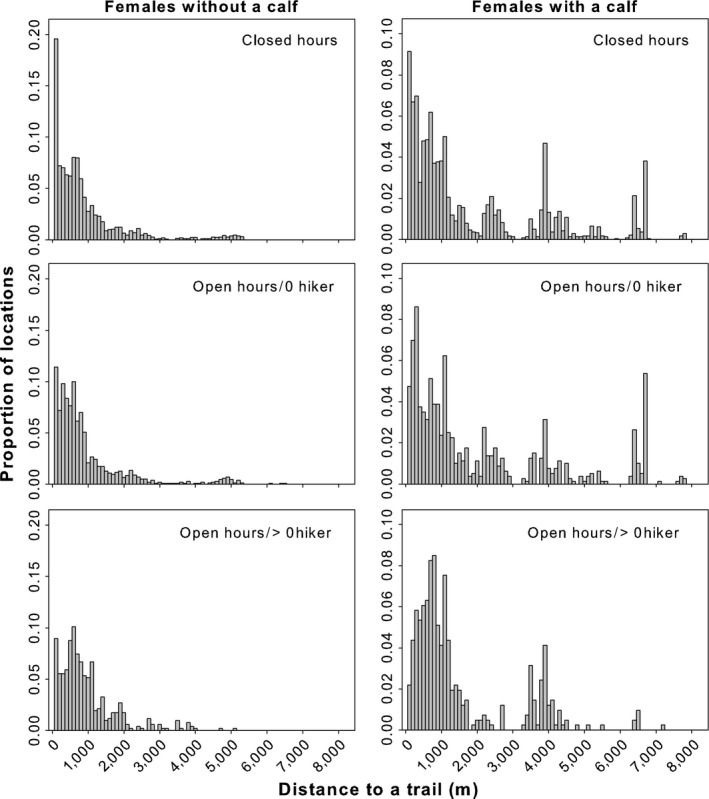
Proportion of locations of female caribou by 100‐m distance classes from a trail in the Gaspésie National Park, during the summers of 2013–2014

We completed 351 focal observations (summer 2013: 9F for 143 focal observations; summer 2014: 15F for 208 focal observations). In 2014, calves suffered a high mortality rate, resulting in a relatively small sample size for that class (i.e., females with a dependent calf) (Table 1). On average, focal observations occurred for 1,728 s (SD = 232). We retained only observations longer than 1,000 s (*n* = 259). This threshold avoided bias that might be associated with dominant behaviors that occur exclusively during short observation periods. We retained 60 and 161 focal observations of females with and without a calf, respectively. On average, females with and without a calf were within 100 m of a trail 7 and 11% of their time, respectively, with a greater use of these areas during closed hours (17:00 to 10:00) (Figure 2). Caribou were further from the trail during hours when the trails were open, especially when hikers were present (all combinations of opening hours/number of hikers for caribou distribution near trails (>4,000, 3,000, 2,000, 1,000 m) were significantly different *p* < .05, except for the distance category <1,000 m for females with a calf; there was no statistically significant difference between closed and open hours when hikers were not present, *p* = .07) (Figure 2).

**Figure 3:**
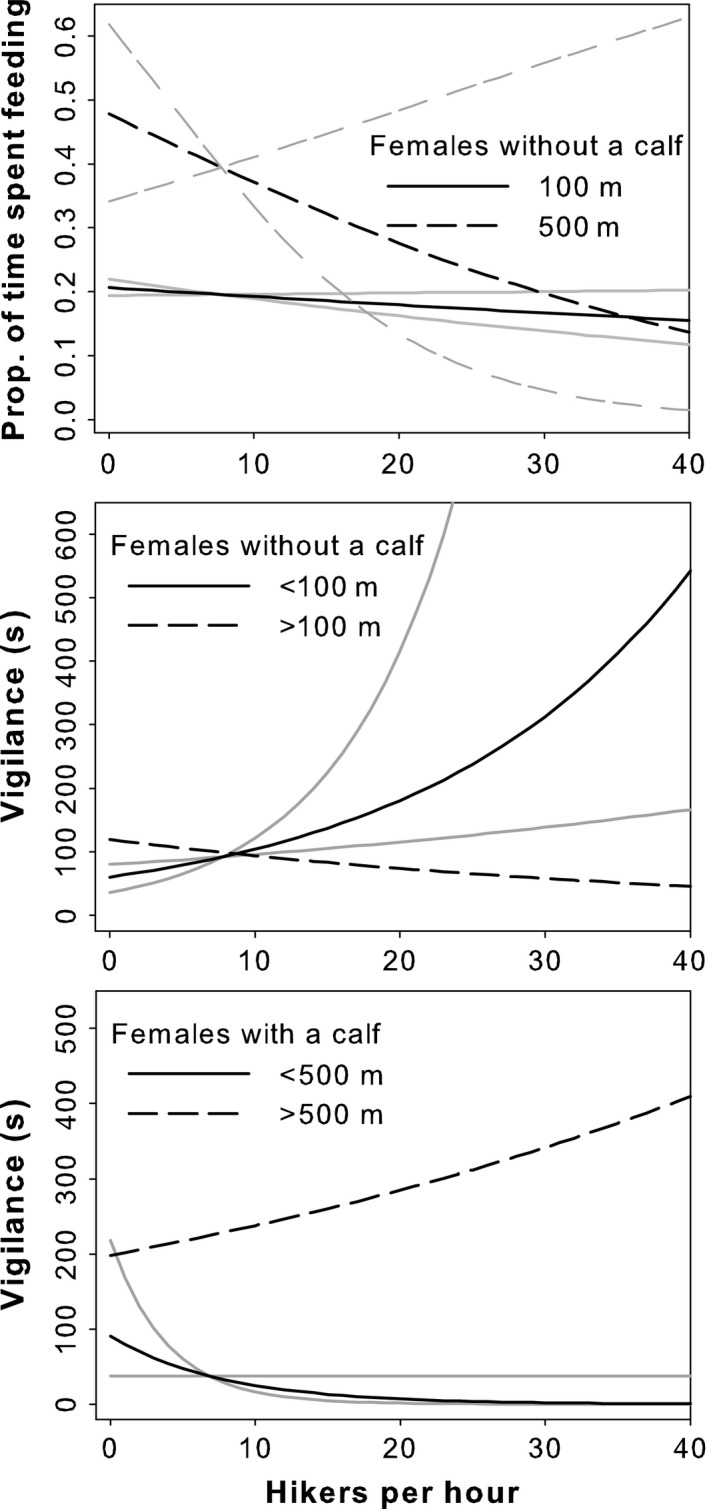
Representation of the most parsimonious models explaining caribou behavior (feeding and vigilance) in relation to their distance to a trail and the number of hikers in the Gaspésie National Park, during the summers of 2013–2014. Gray lines represent the 95% confidence interval

Females had different tolerance to human disturbance as the model selection for distance covariate showed. Caribou with a calf spent a greater amount of time feeding and displaying vigilance behaviors (Table 2), mainly at the expense of time spent lying, although the variability in time spent feeding and being vigilant was very high. The negative binomial and fractional logit models explained a relatively small part of the variation in observed behavior (Spearman's *r* from cross‐validation varied between 0.022 and 0.258) (Table 3). Models that included anthropogenic variables were the most parsimonious in explaining the total time of vigilance for all females and the proportion of time spent feeding for females without a calf (Table 2). Interestingly, vigilance of females with and without a calf differed in their response to human presence (Table 3; Figure 3). The interaction between “Distance to a trail” and “Hikers” revealed that females without a calf increased their vigilance rate near trails relative to the number of hikers, while females accompanied by their calf decreased their time being vigilant under the same circumstances. A posteriori analyses showed that this decrease in vigilance was generally associated with a higher foraging rate (~20%).

**Table 2:** The influence of the presence of a calf and the distance to a trail on the four main behaviors [mean (SD)] of female caribou in the Gaspésie National Park, during the summers of 2013–2014

Behavior	F. with calf				F. without calf			
	<100 m (n = 9)	100–500 m (n = 11)	>500 m (n = 40)	Total (n = 60)	<100 m (n = 45)	100–500 m (n = 65)	>500 m (n = 51)	Total (n = 161)
Feeding	42.2 (42.1)	40.8 (42.0)	50.9 (31.2)	47.7 (34.7)	23.7 (33.2)	28.0 (32.0)	35.6 (32.8)	29.2 (32.7)
Lying	37.5 (47.2)	30.1 (40.5)	20.7 (32.4)	25.0 (36.2)	55.6 (45.2)	50.4 (42.7)	40.2 (41.2)	48.6 (43.1)
Vigilance	2.3 (3.65)	8.8 (20.2)	13.6 (15.7)	11.0 (15.8)	6.3 (11.2)	6.5 (12.1)	7.1 (8.7)	6.6 (10.8)
Walking	9.5 (21.4)	3.7 (4.1)	4.2 (7.3)	4.9 (10.1)	4.8 (8.0)	4.8 (8.1)	5.4 (9.8)	5.0 (8.6)

**Table 3:** Candidate models explaining feeding and vigilance behaviors of female caribou during the summers of 2013 and 2014, Gaspésie National Park

Models		F. with calf			F. without calf		
	K^a^	∆AIC_*c*_	LL	*r*_*s*_	∆AIC_*c*_	LL	*r*_*s*_
Feeding							
Temp. + Grp Size + Open hab. + Hour	4	0.000	−40.2	0.022	4.978	−93.0	0.085
Mod 1. + Trail + Hikers + Trail*Hikers	8	5.052	−38.7	0.103	0.000	−87.2	0.193
Vigilance							
Temp. + Grp Size + Open hab. + Hour	4	5.804	−348.5	0.108	0.093	−759.7	0.099
Mod 1. + Trail + Hikers + Trail*Hikers	8	0.000	−341.4	0.241	0.000	−756.3	0.258

The ranking was based on the AIC_*c*_ for each category of females (i.e., with or without a calf). Model number of parameter (K), log‐likelihood (LL), and difference in AIC_*c*_ values (∆AIC_*c*_) are shown. Model performance was assessed using independent cross‐validation (*r*_*s*_).

a Random factor for individual (ID) was included in all models.

**Table 4:** Coefficient and 95% confidence intervals (CI) of the most parsimonious model explaining feeding and vigilance behavior of female caribou in the Gaspésie National Park, during the summers of 2013–2014

	Feeding		Vigilance	
	Fem. with calf	Fem. without calf	Fem. with calf	Fem. without calf
Temperature	−0.224 [−0.775; 0.327]	−**0.632 [**−**6.367;** −**0.871]**	0.078 [−0.352; 0.507]	−0.063 [−0.464; 0.338]
Group size	−0.014 [−0.587; 0.559]	−0.026 [−0.531; 0.478]	−**0.768 [**−**1.317;** −**0.219]**	−0.258 [−0.662; 0.146]
Open habitat	−0.152 [−0.718; 0.414]	**0.579 [0.122; 1.036]**	−0.243 [−0.684; 0.198]	0.015 [−0.415; 0.445]
Hour	0.185 [−0.105; 0.475]	0.171 [−0.053; 0.394]	−0.075 [−0.302; 0.151]	0.091 [−0.156; 0.339]
Dist. to trail	–	**1.879 [0.437; 3.322]**		
Close/Far a trail (1/0)			−**1.784 [**−**2.607;** −**0.960]**	−0.070 [−0.888; 0.747]
Hikers	–	−0.045 [−0.875; 0.785]	0.210 [−0.393; 0.813]	−0.347 [−0.711; 0.016]
Trail*Hikers	–	−0.969 [−2.712; 0.780]	−**1.704 [**−**3.191;** −**0.217]**	**1.140 [0.162; 2.119]**

Coefficients for which the 95% CI did not overlap zero are shown in boldface.

As the group size of caribou increased, the model for females with a calf predicted a decrease in time spent vigilant. This response was not apparent when considering caribou without a calf (Table 3). Increasing temperature influenced the feeding time of females without a calf negatively, while the proportion of open habitat had a positive effect. For both groups of caribou, distance to the nearest trail had a greater influence on feeding time rather than the number of hikers or the interaction between the two terms.

## 4 Discussion

Our results suggest that reproductive status and the perception of predation risk are two important factors that influence how ungulates react to low‐risk disturbances, such as the presence of hikers. For woodland caribou in Gaspésie National Park, the presence of humans on hiking trails triggered a response for all females, but was expressed differently depending on the presence of a calf. Thus, the behavioral responses of caribou were too complex to be classified as one of the two concurrent hypotheses. Consistent with the refuge hypothesis, lactating females were less vigilant along trails that were frequented by hikers, but they still avoided trails during daytime, in support of the predation risk hypothesis. In contrast, females without a calf were less tolerant of human presence, being relatively more vigilant when a large number of hikers were common and moved away from trails during the daytime, as suggested by the predation hypothesis.

Even within a species, individuals do not evaluate risk similarly (Winnie & Creel, 2007). Differences in human avoidance among female caribou suggest that risk is context‐specific. Assuming that females adapt their antipredator strategy to the vulnerability of their offspring, as suggested by Dussault et al. (2012) and Leclerc, Dussault, and St‐Laurent (2014), we might assume that females with a calf experience a different landscape of fear than those without a calf (Leblond, Dussault, Ouellet, & St‐Laurent, 2016). This difference is particularly noticeable in Gaspésie National Park, where the two apex predators, coyotes and black bears, are not known to be efficient predators of adult caribou (Crête & Desrosiers, 1995; Boisjoly, Ouellet, & Courtois, 2010; Bastille‐Rousseau et al., 2016). Our results showed that some lactating females left areas near a trail when hikers were frequent, but those that stayed were less vigilant within the 500‐m threshold distance from trails. This 500‐m threshold is relatively consistent with other studies of ungulates that have reported a human‐induced refuge from predators (Berger, 2007; Shannon et al., 2014). Although coyotes and black bears were found to use trails and gravel roads more often than expected based on random locations in our study area (see Gaudry, 2013; a companion study conducted in the Gaspésie National Park), the automated camera traps distributed along the hiking trails showed that no coyote and only a few black bears used trails in the hour following the passage of a hiker (F. Lesmerises & M.‐H. St‐Laurent, unpublished data). These data suggested that predators avoided humans; similar results were observed in other studies and regions (Ciucci, Boitani, Francisci & Andreoli, 1997; Hebblewhite & Merrill, 2008). In total, this information supports the assertion of human‐induced refuges. The hypothesis of refuge from predator is a plausible explanation for variation in time spent vigilant in relation to hikers. However, we cannot clearly test the mechanisms that might explain that response as we did not consider survival or predator activity inside/outside the refuge. To our knowledge, a similar response has not been reported for other populations of woodland caribou at the behavioral scales we assessed. Yet, many caribou populations suffer from high predation rates across landscapes with human activity (Bradley & Neufeld, 2012; Festa‐Bianchet, Ray, Boutin, Côté, & Gunn, 2011; Whittington et al., 2011), especially on calves (Gustine, Parker, Lay, Gillingham, & Heard, 2006; Leclerc et al., 2014).

Findings from other populations of *Rangifer* also suggest that females with a calf incur a higher nutritional cost associated with gestation during the last trimester and milk production to support the dependent calf (Parker, Barboza, & Gillingham, 2009). Facing higher energetic demands and seeking to maximize nutritional intake, lactating females were probably more prone to accommodate low‐risk disturbances, such as small groups of interspersed hikers (Gustine et al., 2006).

Trade‐offs in predation risk relative to forage intake or quality have been observed for other species that have high nutritional requirements or are in poor condition (Lima & Dill, 1990). From a behavioral perspective, this is often expressed as an altered activity budget where vigilance is decreased to the benefit of increased feeding rates (Winnie & Creel, 2007; but see Hamel & Côté, 2008). As widely observed in other taxa (coati (*Nasua narica*): Burger & Gochfeld, 1992; birds: Lima, 1995; Przewalski's gazelle (*Procapra Przewalskii*): Li et al., 2012), lactating females decrease their vigilance rate with increasing group size, relying more on conspecifics to alert the group of danger. Such adjustment of antipredator tactics in accordance with foraging demands was also found in male elk (*Cervus canadensis*) in Montana (Winnie & Creel, 2007). In our study, it is unlikely that female caribou demonstrated increased foraging activity along trails because of higher forage quality or quantity. We did not specifically test for differences in nutritional quality near and distant to trails, but vegetation sampling did not reveal a systematic difference in vegetation community or greater quantity of forage adjacent to trails (F. Lesmerises & M.‐H. St‐Laurent, unpublished data).

The decrease in vigilance that we observed for lactating females may be a relatively new response by caribou in this population. In contrast, Dumont (1993) working in the same study area observed an increase in the time spent vigilant by females with and without a calf. This difference could potentially be explained by the fact that Dumont (1993) did not consider other covariates in his model and he did not integrate the effect of distance of individual animals to a trail. However, female caribou could also face a higher risk of predation recently as coyote populations are likely increasing in the Gaspésie region (St‐Laurent, Ouellet, Mosnier, Boisjoly, & Courtois, 2009; MFFP unpublished data). Lactating females are now potentially more vulnerable to predation resulting in this use of human‐induced refuge. Alternatively, caribou could have developed some level of habituation to hikers, such as has been observed for other human‐caused disturbances (Stankowitch, 2008; Brown et al., 2012; Johnson & Russell, 2014). Even if these tactics resulted in lower vigilance and higher foraging rates in relatively predator‐free areas, these choices may carry unobserved physiological costs. Other studies have revealed different physiological stresses caused by human presence [e.g., increased cortisol level (Eggermann, Theuerkauf, Pirga, Milanowski, & Gula, 2013), higher heart rate (Weisenberger, Krausman, Wallace, De Young, & Maughan, 1996)] that we did not measure in this study.

During our focal observations, only one coyote was heard and one black bear viewed in the vicinity of caribou. Fresh indirect signs (i.e., tracks and scats) were, however, frequently observed in the morning, suggesting nocturnal movements of predators; this was corroborated by our camera traps along trails. Assuming that predators avoided humans, as suggested by the refuge hypothesis (Berger, 2007; Shannon et al., 2014) and previous studies (Theuerkauf et al., 2003; Hebblewhite & Merrill, 2008; Ordiz et al., 2011; Valeix et al., 2012), caribou should have been found near trails only during the daytime, which was contrary to our results. The location data from the GPS collars revealed that the greatest use of areas near hiking trails by caribou occurred during the night. The similarity in nocturnal distribution of caribou and predator probably increased the encounter rates near trails as reported by Whittington et al. (2011). Despite the diurnal refuge provided by trails, calf survival during the study was extremely low, being no better than survival observed during the study of Dumont (1993) (see Lalonde, 2014 for historical calf recruitment rates), when caribou did not show any use of the same trails.

## 5 Conclusion

Hikers influenced caribou behavior, especially the vigilance–feeding trade‐off. Females without a calf increased their time spent in vigilance near trails and humans, while females accompanied by a calf were more prone to accommodate such relatively low‐risk disturbance. Facing higher predation risk and nutritional demands, some lactating females appeared to use the presence of hikers as a shield against predation. However, given the extremely low calf survival in this population, this adaptation was not sufficient to counterbalance the negative impact of predators that used linear structures at night. These findings highlighted the importance of the appropriate management of linear structures and human activities, especially across the critical habitat of endangered species.

## Data accessibility

Data used to perform this study are available on the Dryad Digital Repository website (doi: http://dx.doi.org/10.5061/dryad.ff6d4).

## Conflict of interest

None declared.
